# Multiply spliced HIV RNA is a predictive measure of virus production *ex vivo* and *in vivo* following reversal of HIV latency

**DOI:** 10.1016/j.ebiom.2021.103241

**Published:** 2021-02-26

**Authors:** Jennifer M. Zerbato, Georges Khoury, Wei Zhao, Matthew J. Gartner, Rachel D. Pascoe, Ajantha Rhodes, Ashanti Dantanarayana, Megan Gooey, Jenny Anderson, Peter Bacchetti, Steven G. Deeks, James McMahon, Michael Roche, Thomas A. Rasmussen, Damian FJ Purcell, Sharon R. Lewin

**Affiliations:** a Department of Infectious Diseases, The University of Melbourne at The Peter Doherty Institute for Infection and Immunity, Melbourne, Australia; b Department of Microbiology and Immunology, The University of Melbourne at the Peter Doherty Institute for Infection and Immunity, Melbourne, Australia; c School of Health and Biomedical Sciences, RMIT University, Melbourne, Australia; d HIV Characterisation Laboratory, Victorian Infectious Diseases Reference Laboratory, the Peter Doherty Institute for Infection and Immunity, Melbourne, Australia; e Department of Epidemiology and Biostatistics, University of California, San Francisco, California, USA; f Department of Medicine, Division of HIV/AIDS, University of California San Francisco, San Francisco, USA; g Department of Infectious Diseases, Alfred Hospital and Monash University, Melbourne, Australia; h Victorian Infectious Diseases Service, Royal Melbourne Hospital at the Peter Doherty Institute for Infection and Immunity, Melbourne, Australia

**Keywords:** HIV, Multiply-spliced HIV RNA, Reservoir, Shock and kill, Latency reversal, Biomarker

## Abstract

**Background:**

One strategy being pursued to clear latently infected cells that persist in people living with HIV (PLWH) on antiretroviral therapy (ART) is to activate latent HIV infection with a latency reversing agent (LRA). Surrogate markers that accurately measure virus production following an LRA are needed.

**Methods:**

We quantified cell-associated unspliced (US), multiply spliced (MS) and supernatant (SN) HIV RNA by qPCR from total and resting CD4+ T cells isolated from seven PLWH on ART before and after treatment *ex vivo* with different LRAs, including histone deacetylase inhibitors (HDACi). MS and plasma HIV RNA were also quantified from PLWH on ART (n-11) who received the HDACi panobinostat.

**Findings:**

In total and resting CD4+ T cells from PLWH on ART, detection of US RNA was common while detection of MS RNA was infrequent. Primers used to detect MS RNA, in contrast to US RNA, bound sites of the viral genome that are commonly mutated or deleted in PLWH on ART. Following *ex vivo* stimulation with LRAs, we identified a strong correlation between the fold change increase in SN and MS RNA, but not the fold change increase in SN and US RNA. In PLWH on ART who received panobinostat, MS RNA was significantly higher in samples with detectable compared to non0detectable plasma HIV RNA.

**Interpretation:**

Following administration of an LRA, quantification of MS RNA is more likely to reflect an increase in virion production and is therefore a better indicator of meaningful latency reversal.

**Funding:**

NHMRC, NIH DARE collaboratory.

**Research in context**

**Evidence before this study**

In people living with HIV (PLWH) on antiretroviral therapy (ART), HIV persists as a long-lived latent form. One strategy being developed to eliminate infected cells that persist on ART is to activate latent virus to produce viral proteins or virions. There are currently no biomarkers or determinants of effective HIV latency reversal following *ex vivo* or *in vivo* treatment with a latency reversing agent (LRA). It is also unknown which marker of HIV persistence best predicts viral protein expression and subsequent immune recognition following a clinical intervention. To date in clinical trials of LRAs, a common primary endpoint for efficacy has been unspliced (US) HIV RNA. Although clinical trials of LRAs in PLWH on ART have shown increases in US RNA *in vivo*, they have not shown any decrease in the frequency of infected cells and have not shown consistent increases in plasma HIV RNA, suggesting that US RNA may not be the best biomarker for latency reversal. Here we sought to determine if multiply spliced (MS) HIV RNA could serve as a biomarker for efficient latency reversal, both *ex vivo* and *in vivo*.

**Added value of this study**

We showed that in PLWH on ART in the absence of any intervention, MS RNA was rarely detected whereas US RNA was always detected. Following stimulation of both resting and total CD4+ T cells from PLWH on ART *ex vivo* with a range of LRAs, the fold change increase in MS RNA correlated with the fold change increase in supernatant (SN) HIV RNA, whereas a similar relationship between US RNA and SN HIV RNA was not detected. In a prospective clinical trial of the LRA panobinostat in PLWH on ART, we found that higher levels of MS RNA were associated with detection of plasma HIV RNA. Finally we showed that detection of MS RNA was not only a measure of increased transcription, but reflected the frequency of intact virus, given the location of primers used to detect MS RNA bind in sites of the viral genome that are commonly mutated or deleted in PLWH on ART.

**Implications of all the available evidence**

Following administration of an LRA, quantification of MS RNA is more likely to reflect an increase in virion production and is therefore a better indicator of meaningful latency reversal.

Alt-text: Unlabelled box

## 1 Introduction

Long-lived and proliferating latently infected CD4+ T cells represent the main barrier to a cure in people living with HIV (PLWH) on antiretroviral therapy (ART) [1], [2], [3], [4]. While ART has significantly extended life expectancy for PLWH, it is not curative [5]. One strategy aimed at eliminating latently infected cells is to activate HIV transcription and virus production to allow for immune mediated clearance or virus induced cytolysis using a latency reversing agent (LRA) [6]. However, it is currently unclear how to best measure latency reversal *ex vivo* or *in viv*o and more importantly, which marker of HIV persistence best predicts viral protein expression and immune recognition following an intervention.

After HIV integration, multiple steps are required to produce infectious virions. Initiation of viral transcription from the integrated provirus is followed by efficient m7G-capping and processive elongation [7] to form unspliced (US) HIV RNA, which ultimately gets packaged in a virion. At the same time, co-transcriptional splicing occurs to form singly spliced (SS; meaning only one splice site) and multiply spliced (MS; meaning more than one splice site) RNAs [8,9]. Translation of MS RNA produces regulatory proteins including trans-activator of transcription (Tat), Rev, and Nef [10]. Tat localises to the nucleus and enhances the efficiency of full proviral transcription and RNA-processing leading to an increase in translation more than 1000-fold [11]. The HIV Rev protein assists in export of RNA to the cytoplasm and translation of US and SS mRNAs through binding their Rev-responsive element [12], [13], [14]. Translation of US RNA gives rise to Gag and Gag-Pol polyproteins, whereas translation of SS HIV RNA produces Env and the accessory proteins Vif, Vpu, and Vpr [15]. Therefore, the relative amounts of US, SS, MS RNA as well as Tat and Rev will have a profound effect on the efficiency of virus production from an infected cell.

Traditionally, latently infected cells have been described as being transcriptionally silent. However, it has been known for over two decades that US RNA is nearly always detected in CD4+ T cells collected from blood from PLWH on ART [16]. Furthermore, we previously demonstrated that following the administration of the HDACi vorinostat *in vivo*, a larger fold increase in US RNA post-vorinostat was associated with a higher basal level of US RNA [17]. Therefore, the basal constitutive expression levels of US RNA may be indicative of the likelihood of increased initiation of transcription following an LRA, either *ex vivo* or *in vivo*. However, a change in US RNA may not result in successful virus production given that the process of HIV transcription initiation, capping, elongation, splicing and termination occurs inefficiently in latently infected CD4+ T cells in the blood [18] and in the gastrointestinal tract [19]. Others have also shown that LRAs such as HDACi are unable to induce viral elongation or splicing [18,20].

Evaluation of LRAs that modulate transcription in clinical trials of PLWH on ART have demonstrated an increase in the initiation of HIV transcription from an integrated provirus, measured as an increase in cell-associated US HIV RNA, but varying efficacy in increasing plasma HIV RNA [6]. In clinical trials of the histone deacetylase inhibitors (HDACi) vorinostat [17,21,22] and the CCR5 antagonist maraviroc [23], an increase in US HIV RNA was observed with no significant change in plasma HIV RNA. The HDACi romidepsin led to an increase in both US and plasma HIV RNA in some [24,25] but not all studies [26]. The anti-alcoholism drug disulfiram, when administered at higher doses induced a modest increase in both US and plasma HIV RNA [27,28].

Toll like receptor agonists can also reverse HIV latency with varying efficacy in human and non-human primate studies. The toll like receptor (TLR)-9 agonist induced an increase in plasma HIV RNA but with no change in US RNA [29,30]. In a non-human primate model using simian immunodeficiency virus (SIV) or SIV containing an HIV envelope (SHIV), significant increases in plasma SIV RNA were observed following the repeated administration of the TLR-7 agonists GS-986 or GS-9620 in some but not all studies [31], [32], [33]. In PLWH on ART, a dose-escalation, placebo-controlled study of the TLR7 agonist Vesatolimod (GS-9620) had no effect on the cell-associated HIV DNA, RNA, or plasma viral load [34]. It is currently unclear what biomarker can predict effective latency reversal *in vivo*, and which LRAs are able to induce production of HIV proteins and virions from latently infected cells.

In this study, we measured US, MS and supernatant (SN) HIV RNA from both total and resting CD4+ T cells isolated from blood from PLWH on ART following stimulation with different LRAs. Using a database of published HIV proviral sequences derived from CD4+ T cells from PLWH on ART [35], we showed that the target sequence for primers designed to detect MS RNA compared to US RNA more commonly contained deletions and mutations and therefore detection of MS RNA does not just reflect transcriptional activity of the cells but also partly reflects expression of RNA from an intact virus. We found that all LRAs led to an increase in US RNA, except for vorinostat, and to a lesser extent an increase in MS RNA, but had little to no effect on SN RNA. Moreover, the fold increase in MS RNA strongly correlated with the fold increase in SN RNA, whereas the fold increase in US RNA did not. Following the administration of panobinostat to PLWH on ART, detection of plasma HIV RNA occurred more frequently in samples with higher levels of MS RNA in CD4+ T cells. Following administration of an LRA, quantification of MS RNA is more likely to reflect an increase in virion production and is therefore a better indicator of meaningful latency reversal.

## 2 Methods

### 2.1 Ethics

The use of blood samples from HIV negative donors for this study was approved by the Human Research and Ethics Committees from the Alfred Hospital (HREC156/11), Monash University (CF11/1888) and the University of Melbourne (1443071). Adult donors were recruited by the Red Cross Blood Transfusion Service and all provided written informed consent for the use of their blood products for the research. The use of blood samples from adult PLWH was approved by the Alfred Hospital (HREC214/15) for the study entitled Large volume peripheral blood mononuclear cells (PBMC) collection by leukapheresis to define HIV persistence in HIV-infected adults. Adult PLWH were recruited with viral loads <50 copies per mL for at least 2 years. All participants provided informed consent and the protocol was approved by the local Institutional Review Board. For the prospective panobinostat clinical trial, adult PLWH were enrolled with viral loads <50 copies per mL for at least 2 years. Additional details can be found at ClinicalTrials.gov number NCT01680094 or as previously described [35]. All participants provided informed consent and the protocol was approved by the local Institutional Review Board.

### 2.2 Participant details

PLWH on ART with a viral load <20 copies/ml for ≥3 years were eligible for enrolment. Peripheral blood mononuclear cells (PBMCs) were collected by leukapheresis (Alfred Hospital, Melbourne, Australia) and stored in liquid nitrogen. In a separate study, PLWH on ART were enrolled in a prospective single arm study of intermittent panobinostat 20 mg three times a week, every other week for 8 weeks ([36]; ClinicalTrials.gov number NCT01680094).

### 2.3 Ex vivo studies of total and resting CD4^+^ T cells from PLWH on ART

PBMCs were thawed and either total or resting CD4^+^ T cells were isolated by negative selection using the EasySep™ Human CD4+ T Cell Isolation Kit or the EasySep™ Human Resting CD4+ T Cell Isolation Kit, respectively (StemCell Technologies, cat # 17952 and 17962, respectively). Cells were cultured at 5 million cells per well in a 24-well plate in RPMI-1640 (Life Technologies, cat # 21870092) supplemented with 10% FBS (Interpath) and pen/strep/glut (Life Technologies, cat # 10378016). Cells were stimulated with DMSO (1:2000, Sigma Aldrich, cat # D2650-5 × 10 ML), vorinostat (500 nM, Selleckchem, cat # S1047), romidepsin (40 nM, Selleckchem, cat # S3020), panobinostat (30 nM, Selleckchem, cat # S1030), JQ1 (1 µM, Selleckchem, cat # S7110), or PMA+PHA (10 nM, Sigma Aldrich, cat # P8139-1 MG; 10 μg/mL, Thermo Fisher Scientific, cat # R30852701), all in the presence of raltegravir (1 µM, Selleckchem, cat # S2005) and IL-2 (1 U/mL, Sigma Aldrich, cat # 10799068001) for 3 days. For the panobinostat study, frozen PBMCs were thawed and total CD4+ T cells were isolated via negative selection using the CD4+ T Cell Isolation Kit, Human (Miltenyi Biotec, cat # 130-096-533).

### 2.4 Cell-associated unspliced and multiply spliced HIV RNA qPCR assays

After 3 days, cells were harvested in Trizol (Life Technologies, cat # 15596018). Cell-associated RNA was extracted following the manufacturer's protocol and the RNA was then DNase-treated with RQ1 DNase (Promega, cat # M6101). 500 ng of RNA was reverse transcribed into cDNA using random hexamers (Life Technologies, cat # N8080127), oligo(d)T (Life Technologies, cat # 18419012), and Superscript III reverse transcriptase (Life Technologies, cat # 18080085). 125 ng of cDNA per condition was then used to perform a semi-nested real time quantitative (q) PCR assay for US RNA as previously described [17,37]. MS RNA was also measured using a semi-nested qPCR assay using the primers outlined in Supplementary Table 2, as described previously [37]. The limit of detection for both the US and MS qPCR assays was 1 copy per well. All samples were run in quadruplicate with two no RT control wells to assess for DNA contamination. In order to calculate the fold change from DMSO control, all replicate wells that were undetectable or below 1 copy, were assigned a value of 0.5 copies. For *ex vivo* HIV RNA data, HIV RNA was normalized per RNA input, measured by NanoDrop (ThermoFisher), and presented as copies per 500 ng RNA. For the prospective panobinostat clinical trial, HIV RNA was normalized to the 18s housekeeping gene using the 18s Taqman gene Expression Assay (Life Technologies, cat # 4331182) and HIV RNA is presented as copies per 10^6 18s copies, as previously described.

### 2.5 Quantification of supernatant HIV RNA

After 3 days, culture supernatant was harvested by centrifugation at 1000 x g for 10 min to remove cells and debris. 1 mL of supernatant from each condition was then run on the COBAS® AmpliPrep/COBAS® TaqMan HIV test kit v2.0 for the quantification of HIV RNA (Roche Diagnostics). Culture supernatants assessed using this method have previously been shown to be free of contaminating HIV DNA [38]. The limit of quantification for this assay is 20 copies/mL so anything that was undetectable or <20 was assigned a value of 20 copies/mL.

### 2.6 HIV DNA qPCR assay

Total cellular DNA was extracted using the Qiagen AllPrep kit (Qiagen, cat # 80204). Total HIV DNA was quantified as described previously [39] and normalized to the number of cell input as measured by the *CCR5* gene, as previously described [40].

### 2.7 Analysis of defective proviral sequences for primer binding sites

Defective proviral sequences were obtained using the HIV Proviral Sequence Database [35]. All HIV RNA primers and proviral sequences were aligned to the HXB2 reference genome using CLC genomics workbench v10 (Qiagen). Only defective proviral sequences isolated from the blood of PLWH suppressed on ART with a viral load <50 copies/mL were analyzed. 993 sequences were obtained at the time of query. Primer sequences were considered as mutated if the sequence was present but one or more base pair mutation was present. Primer sequences were considered deleted if the sequence was not present at all. Primer sequences were considered as a perfect match if the sequence was present and did not contain any mutations or deletions.

### 2.8 Flow analysis of T cell proliferation and death

Total and resting CD4+ T cells were isolated as described previously from buffy backs from three HIV-uninfected individuals from the Red Cross blood transfusion service. CD4+ T cells were plated at 500,000 cells per well in a 96-well plate and stimulated with LRAs for 1, 3 and 6 days as described above. Following stimulation, cells were stained with live/dead cell viability dye (Life Technologies, cat # L34955) for 15 min at room temperature followed by surface staining for CD3-BV711 (BD Biosciences, cat # 563725, RRID:AB_2744392) and CD4-APC (BD Biosciences, cat # 555349, RRID:AB_398593) on ice for 30 min. Cells were then intracellularly stained for Ki-67-PE (BD Biosciences, cat # 556027, RRID:AB_2266296) using the BD Cytofix/Cytoperm Fixation/Permeabilization Solution Kit (BD Biosciences, cat # 554714) according to the manufacturer's protocol. Flow cytometry was assessed on an LSRFortessa (BD Biosciences) and data were analyzed using FlowJo v.10.

### 2.9 Statistical analyses

**Fig. 1:**
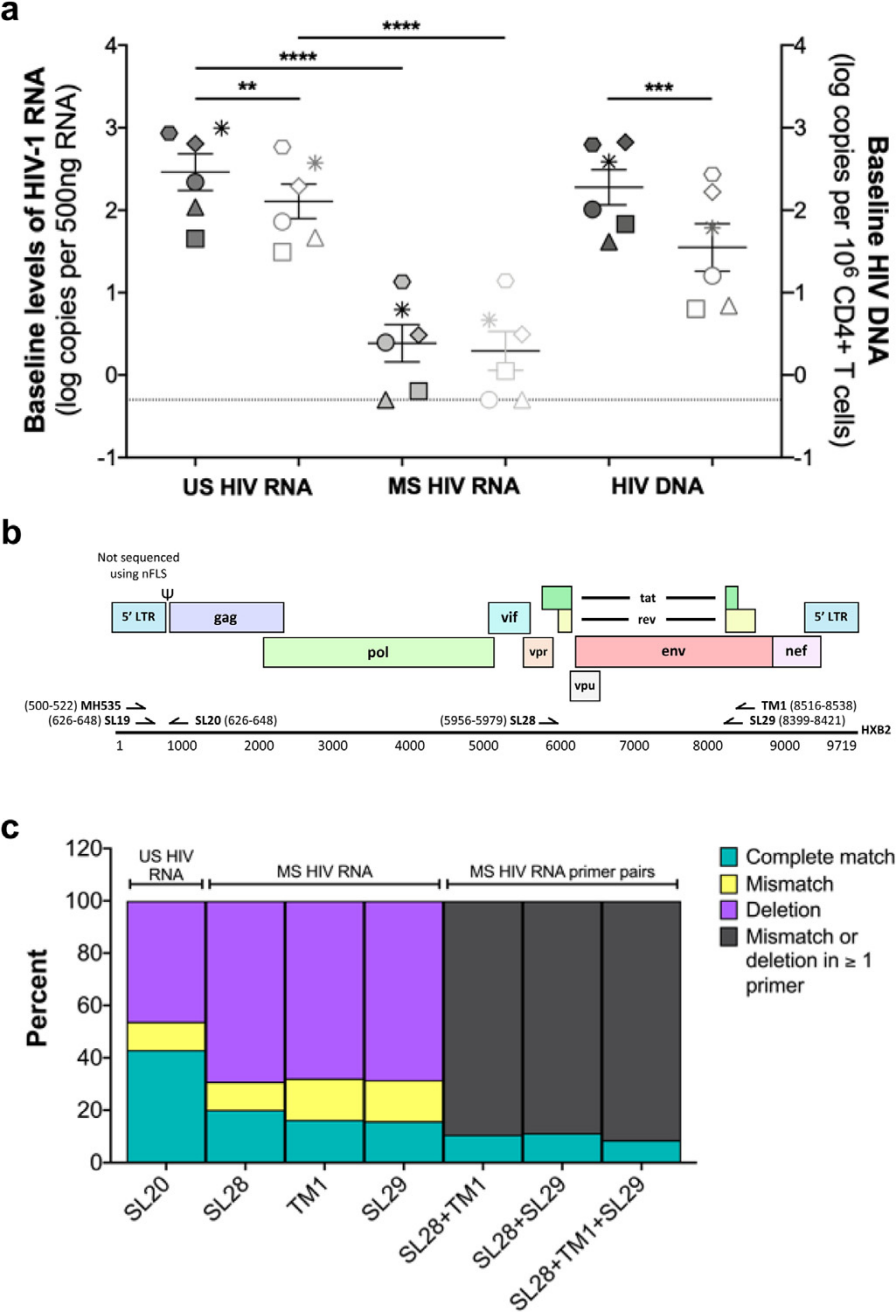
**Expression of US and MS RNA and HIV DNA in total and resting CD4+ T cells isolated from PLWH on suppressive ART.** (**a**) US and MS HIV RNA and HIV DNA were quantified in freshly isolated total (solid symbols) and resting (open symbols) CD4+ T cells. p values were determined by paired t test on logarithmically transformed data. All data are shown as the mean +/- SEM. Each symbol represents a single donor. N = 6. *p < 0.05; **p <  0.01; ***p < 0.001; ****p < 0.0001. (**b**) Schematic alignment of US and MS RNA primer sequences in reference to the HIV genome and HXB2 sequence. HIV DNA quantification was a single PCR amplification and used SL19 and SL20 (**c**) The percent of each primer or primer pair that is a complete match, contains a base pair mismatch(es) or is deleted in 993 representative defective proviral sequences obtained from the proviral sequence database [35].

**Fig. 2:**
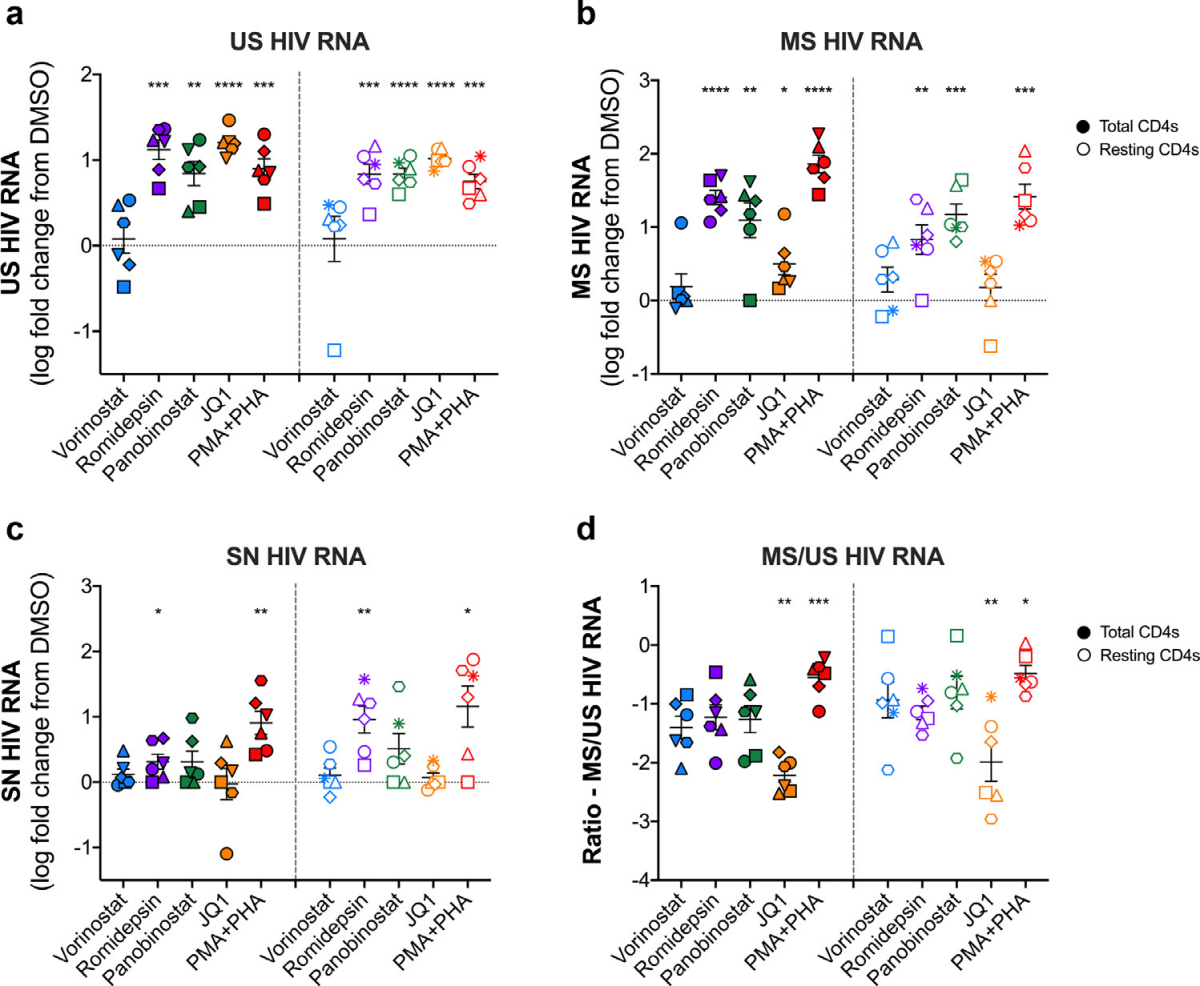
**Changes in US, MS and SN RNA following stimulation of total and resting CD4+ T cells with LRAs after 3 days.** Total and resting CD4+ T cells from PLWH on ART were stimulated for 3 days with different LRAs and (**a**) US RNA and (**b**) MS RNA were quantified. The fold change from DMSO at day 3 is shown. (**c**) The ratio of MS/US RNA was calculated using the raw RNA copy numbers. (**d**) SN RNA was quantified 3 days following stimulation with different LRAs. All data are shown as the mean +/- SEM. Each symbol represents a single donor. N = 6. p values were calculated using a one sample t test on logarithmically transformed data comparing each LRA to the DMSO control. *p < 0.05; **p < 0.01; ***p < 0.001; ****p < 0.0001.

**Fig. 3:**
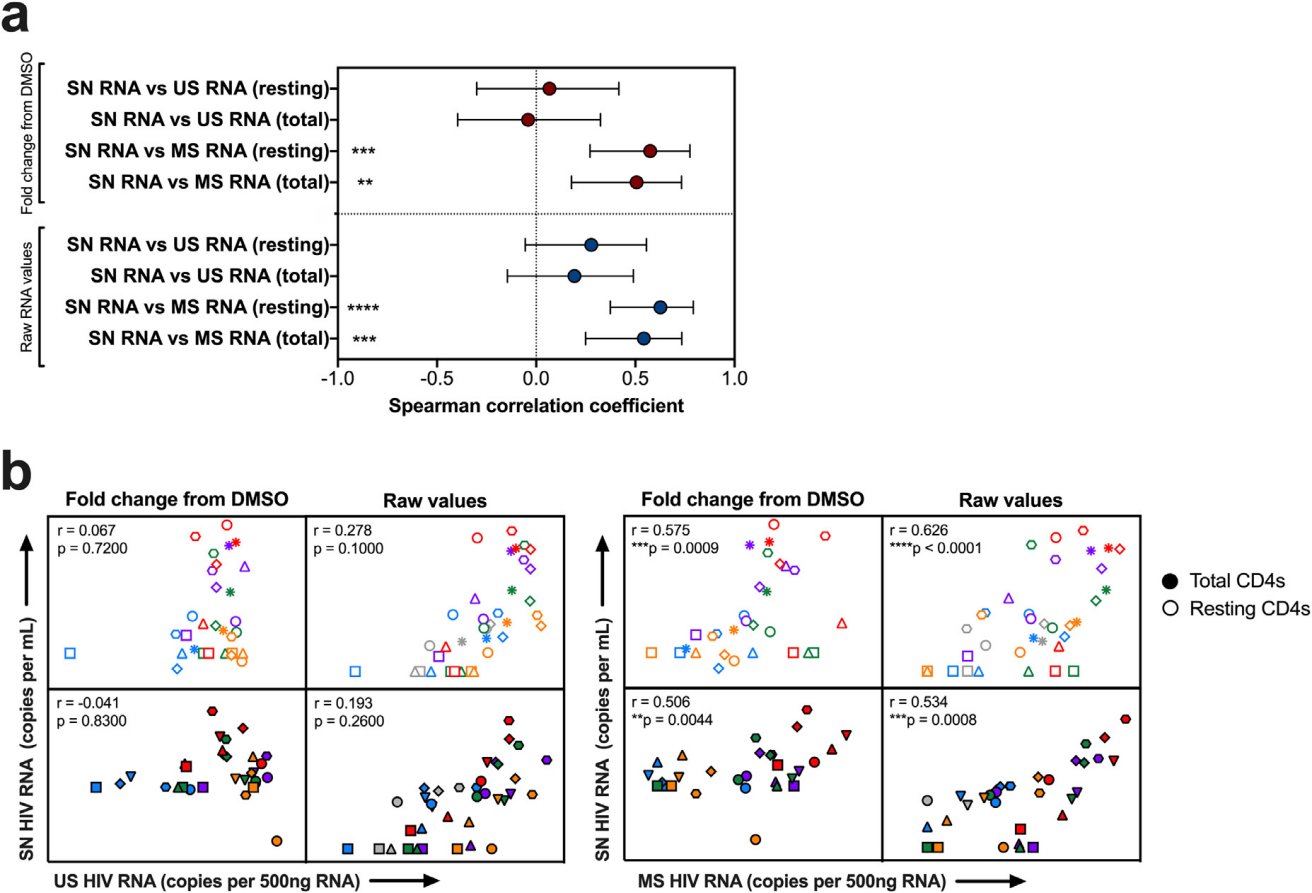
**Correlation between SN RNA and either US or MS RNA 3 days post-stimulation with LRAs.** (**a**) Spearman correlation coefficients and 95% confidence intervals are shown for comparisons between SN RNA and US or MS RNA in total and resting CD4+ T cells after adjustment for repeated measures from the same donor using raw and fold change data following stimulation with LRAs. (**b**) Scatter plots are shown for the fold change from DMSO and the raw values of each parameter quantified in total (closed) and resting (open) CD4+ T cells. The Spearman correlation coefficients (r) and p values after controlling for multiple observations per donor are shown. Each donor is shown as a different symbol and each LRA a different color. Gray = DMSO, blue = vorinostat, purple = romidepsin, green = panobinostat, orange = JQ1, PMA+PHA = red. *p  < 0.05; **p < 0.01; ***p < 0.001, ****p < 0.0001.

**Fig. 4:**
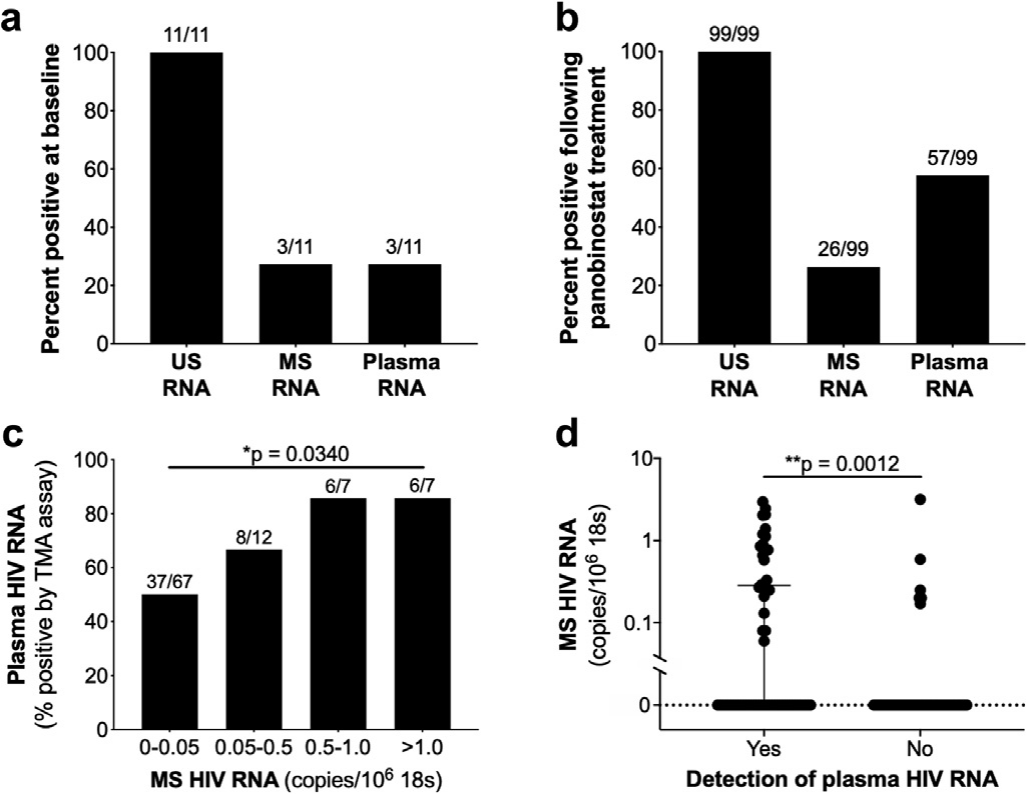
**Detection of MS RNA is associated with detection of plasma HIV RNA following administration of the HDACi panobinostat to PLWH on ART. (a)** US, MS, and plasma HIV were measured and the percentage of participants positive for each RNA measure are shown for timepoints prior to panobinostat and (**b**) on panobinostat treatment. (**c**) The proportion of participants with a positive plasma HIV RNA according to the amount of MS RNA. (**d**) Amount of MS RNA in samples collected at time points prior to and following panobinostat where plasma HIV RNA was detected or not . For d, data are shown as median +/- IQR. *p  < 0.05; **p < 0.01; ***p < 0.001; ****p < 0.0001*.*

All statistical analyses were performed using GraphPad Prism v7.0 or SAS version 9.4. All statistical comparisons were performed on logarithmically transformed data except for Fig. 4d. Paired t tests were used to compare baseline RNA and DNA measurements (Fig. 1a). A one sample t test was used to compare the fold change in RNA following LRA treatment compared to DMSO (Fig. 2). Paired t tests were used to compare RNA measurements versus DMSO control (Supplementary Figure 2). Simulation studies have shown that the t-test is robust to non-normality and small sample sizes. (see https://scholarworks.umass.edu/cgi/viewcontent.cgi?article=1307&context=pare As there are no principal objections to using a t-test with Ns as small as 2, we preferred using t-tests because Wilcoxon signed-rank tests are unable to incorporate quantitative information. In addition, the logarithmic transformation that we have used has largely resulted in approximate normality for these measures in separate larger studies. While outliers can disrupt t-tests, we did not appear to have significant outliers in our data sets. Pearson correlations with 95% confidence intervals were calculated to compare baseline RNA to day 3 RNA (Supplementary Figure 3a-b). Multiple observations from the same donor (six different conditions) are not independent observations, so we modified analyses for correlations involving multiple observations per donor. We first obtained residuals from a model of each RNA measurement that controlled for donor. We then examined Spearman correlations among the models’ residuals for the pairs of RNA measurements of interest (Fig. 3a). Scatter plots (Fig. 3b) show the data, with different symbols indicating multiple observations from each donor. The correlation coefficients and p values shown are adjusted for multiple observations per donor. Comparisons between non-normally distributed data were made using a Mann-Whitney test (Fig. 4d). P values < 0.05 were considered statistically significant.

### 2.10 Role of funding source

The funding sources had no role in the study design, data collection, data analysis, interpretation, writing, or decision to publish. The authors have not been paid to write this manuscript by any agency or funding body.

## 3 Results

### 3.1 Defective proviruses may partly explain the significantly lower levels of MS RNA compared to US RNA in PLWH on ART

We first measured HIV DNA and US and MS RNA levels in total and resting CD4+ T cells isolated from PLWH on ART (N=7, where 5 donors had both total and resting cells collected and 1 donor had only total and the other donor only resting cells isolated; Supplementary Table 1). At baseline, HIV DNA and US RNA were significantly higher in total versus resting CD4+ T cells (HIV DNA median: 245 vs 39 copies/million cells, respectively, *p* = 0.0005; US RNA median: 429 vs 139 copies per 125 ng, respectively, *p* = 0.0025, [paired t test]) (Fig. 1a). MS RNA levels were low in total and resting CD4+ T cells (median 2.8 vs 2.1 copies per 125 ng, respectively, *p* = 0.5059, [paired t test]) (Fig. 1a). These data show that the basal transcriptional activity was higher in total CD4+ T cells compared to resting CD4+ T from PLWH on ART but both cell types had low or no detectable MS RNA.

Given that in PLWH on ART ~95% of proviruses in circulating CD4+ T cells are defective [41], [42], [43], [44], [45], [46], and defects have been commonly described in the first Tat coding exon [47], we wanted to determine whether the frequency of these defects differed between the sequences targeted by primers that we used to detect US and MS RNA transcripts (Supplementary Table 2). If defects were more common for example in the target sequences for the MS RNA primers, then detection of this MS RNA product could be a surrogate indicator of efficient transcription from an intact provirus.

We analysed 993 defective proviral sequences obtained via near full-length sequencing (nFLS) of CD4+ T cells from PLWH on ART (https://psd.cancer.gov/; [35]). We determined the percentage of published sequences where the primer binding sites used in the US and MS RNA quantitative PCR (qPCR) assay were either deleted or mutated, with the expectation that these deleted or mutated variants would not be detected in the qPCR assays. The US and MS RNA assays are semi-nested requiring three primer sequences for each (summarised in Supplementary Table 2 and shown in Fig. 1b). It is important to note that the published nFLS does not include the 5’ LTR [41,42,46] and therefore the forward primer MH535 for the first round amplification of US RNA could not be assessed. The forward primer SL19 used for the second round US RNA amplification had 100% sequence overlap to the nFLS BLOuterF primer used for all of the nFLS obtained in the proviral sequence database [41], [42], [43], meaning that every sequence that had been amplified contained this primer binding site (Fig. 1b). Finally, the reverse primer SL20 (used for both first and second round qPCRs for US RNA) bound to a target sequence that was a complete match in 43% of defective proviral sequences in the database. SL19 and SL20 were also used for detection of HIV DNA as a single round qPCR (Supplementary Table 3).

We next evaluated the three MS RNA primers, individually and in pairs for the first round and second round amplification. We found that each primer individually had complete matches to 16-20% of defective proviruses (Fig. 1c). When we looked at primer pairs for first round and second round amplification, we found that the primer pairs had complete matches to 10.7% and 11.3% of defective proviruses, respectively (Fig. 1c). When we looked at all three MS RNA primers, which are required for MS RNA detection, these primers had a complete match with only 8.6% of defective proviruses (Fig. 1c). Furthermore the MS primers preferentially target the *tat* and *rev* subset of 2 kb spliced mRNAs that are essential for efficient expression of HIV regulatory proteins. Taken together, these data demonstrated that a high frequency of defective viruses could alone account for failure to detect MS RNA, independent of transcriptional activity of a cell. Therefore when an MS RNA product is detected, this will be partly a reflection of both the frequency of intact viruses present, in addition to transcription, splicing and degradation of the HIV RNA.

### 3.2 All LRAs increased US RNA except for vorinostat, but only romidepsin, panobinostat and PMA+PHA led to an increase in MS RNA and SN RNA ex vivo

We then stimulated both total and resting CD4+ T cells from PLWH with a panel of LRAs, including HDACi vorinostat, romidepsin, panobinostat, the bromodomain inhibitor JQ1, and PMA+PHA for 3 days. All LRAs were resuspended in DMSO and thus all results were compared to a DMSO only control. We focused on LRAs that do not cause significant T cell activation or cellular proliferation, and included PMA+PHA as a positive control (Supplementary Figure 1). With continuous exposure to romidepsin and panobinostat for 6 days, we found substantial cell death (Supplemental Figure 1b-c). Therefore, we chose to focus our studies on day 3 post-treatment with LRAs only. After 3 days post-treatment with LRAs, cells were harvested for US and MS RNA and supernatant was collected for HIV RNA quantification (Fig. 2, Supplementary Figure 2). The absolute numbers of each variable (Supplementary Figure 2) and fold change over DMSO was quantified (Fig. 2). There was a statistically significant increase in US RNA following treatment with romidepsin, panobinostat, JQ1 and PMA+PHA for both total (*p* = 0.0002, *p* = 0.0019, *p* < 0.0001 and *p* = 0.0005, respectively, [one sample t test], Supplementary Figure 2a) and resting CD4+ T cells (*p* = 0.0008, *p* < 0.0001, *p* < 0.0001 and *p* = 0.0003, [one sample t test], respectively, Supplementary Figure 2a). The highest increases in US RNA for total CD4+ T cells were seen following treatment with romidepsin and JQ1 (16.8 and 15.8 median fold increase over DMSO, respectively, (*p* = 0.0002 and <0.0001, respectively, [paired t test], Fig. 2a)). In resting CD4+ T cells, similar changes were seen with romidepsin, panobinostat, JQ1, and PMA+PHA (ranging from 5.4–9.8 median fold increase over DMSO, *p* = 0.0008, *p* < 0.0001, *p* < 0.0001 and *p* = 0.0003, respectively, [paired t test], Fig. 2a)), consistent with efficient HIV transcription initiation induced by all the LRAs except vorinostat (1.9 median fold increase over DMSO, *p* = 0.7759, [paired t test], Fig. 2a).

There was a statistically significant increase in MS RNA following stimulation with romidepsin, panobinostat, JQ1 (for total CD4+ T cells only (*p* = 0.0216)) and PMA+PHA in total (*p* < 0.0001 for all conditions) and resting (*p* = 0.0089, *p* = 0.0089 and *p* = 0.0004, respectively) CD4+ T cells. (Supplementary Figure 2b). Consistent with these findings, romidepsin, panobinostat, and PMA+PHA induced significant increases in SN RNA in both total (*p* = 0.0419, *p* = 0.0419 and *p* = 0.0038, respectively, [paired t test]) and resting (*p* = 0.0056, *p* = 0.0056, and *p* = 0.0139, respectively, [paired t test]) CD4+ T cells (Fig. 2C, Supplementary Figure 2c). The ratio of MS to US RNA increased relative to DMSO, only following stimulation with PMA+PHA in both total (ratio = 0.34 and *p* = 0.0145, [paired t test]) and resting (ratio=0.43 and p=0.0081, [paired t test]) CD4+ T cells (Fig. 2d). Interestingly, the ratio of MS to US RNA decreased relative to DMSO following stimulation with JQ1 in both total (*p* = 0.0284, [paired t test]) CD4+ T cells and resting (*p* = 0.0585, [paired *t* test]) CD4+ T cells (Fig. 2d).

In summary, we found that initiation of transcription (as measured by US RNA) occurred *ex vivo* with all LRAs except vorinostat. Production of MS RNA was observed following stimulation with panobinostat, romidepsin and PMA+PHA, which was consistent with observed increases in SN RNA.

### 3.3 Positive correlation between the fold change MS and SN HIV RNA following *ex vivo* stimulation with LRAs

We next looked at correlations between US, MS, and SN HIV RNA following stimulation of either total or resting CD4+ T cells with a panel of LRAs and accounted for the possible lack of independence among the multiple observations from the same donor (see methods). We observed a statistically significant correlation between MS and SN RNA for both total and resting CD4+ T cells (*p* = 0.0008 and <0.0001 respectively, [Spearman correlation], Fig. 3a) but no correlation between US and SN RNA for both total and resting CD4+ T cells (*p* = 0.2600 and *p* = 0.1000, respectively, [Spearman correlation], Fig. 3a). Scatterplots (Fig. 3b) show the data, with different symbols indicating the multiple observations from each donor. Spearman correlation coefficients are adjusted for the multiple observations per donor.

We also wanted to know if baseline US or MS RNA was a predictive measure of US or MS RNA production post-treatment with different LRAs. We found strong and highly consistent correlations between US RNA at baseline and at day 3 post-treatment with each LRA for both total and resting CD4+ T cells from PLWH on ART (Pearson correlation coefficients from 0.856 to 0.988, [Pearson correlation], Supplemental Figure 3a, c). Conversely, we did not find statistically significant correlations between MS RNA at baseline and at day 3 post-treatment with each LRA for both total and resting CD4+ T cells from PLWH on ART (Pearson correlation coefficients from 0.166 to 0.835, *p* = 0.7500 to *p* = 0.0780, [Pearson correlation], Supplemental Figure 3b, c) with the exception of treatment with vorinostat and JQ1 in total CD4+ T cells (Pearson correlation coefficients 0.929 to 0.914, *p* = 0.0225 to *p* = 0.0298, respectively, [Pearson correlation], Supplemental Figure 3b, c).

We also evaluated the relationship between fold change from DMSO in each of the viral parameters, which we propose better reflects the activity of the LRA, as this parameter will adjust for the constitutive levels of transcription at baseline in each participant. We observed a statistically significant correlation between the fold change in MS RNA and SN RNA for both total and resting CD4+ T cells (Spearman correlation coefficients 0.501 and 0.489, *p* = 0.0048 and 0.0061 respectively, [Spearman correlation], Fig. 3a). There was no statistically significant correlation between the fold change in US RNA and SN RNA for both total and resting CD4+ T cells (Spearman correlation coefficients 0.123 and 0.147, *p* = 0.52 and 0.44, respectively, [Spearman correlation], Fig. 3a).

### 3.4 Increased MS HIV RNA observed following administration of the HDACi panobinostat to PLWH on ART

To determine if a change in MS RNA and plasma virus following an LRA was also observed *in vivo*, we quantified MS RNA in CD4+ T cells isolated from blood from 11 PLWH on ART who received panobinostat as part of a prospective clinical trial which has been previously reported (NCT01680094) [36]. In this clinical trial, US RNA had been measured using a qPCR assay identical to the one used here in this study, while plasma HIV RNA was measured using a non-quantitative transcription-mediated amplification assay (TMA) where samples were designated as positive or negative [48]. We compared plasma HIV RNA and MS RNA from 11 of the original study participants.

At baseline, US RNA was detected in blood from all participants, whereas only 27.3% of participants had detectable MS or plasma HIV RNA (Fig. 4a). Following panobinostat treatment, combining all data from 11 participants across 10 time points (noting that not all donors had samples at every time point), US RNA was still detected in all participants at all time points, whereas MS and plasma HIV RNA was detected in 26.3% and 57.6% of samples, respectively (Fig. 4b). We next looked at the quantity of MS RNA and the percent of samples positive for plasma HIV RNA combining samples collected prior to and following panobinostat. We found a clear relationship between the amount of MS RNA quantified and the likelihood of detecting plasma HIV RNA (Fig. 4c). With an increased quantity of MS RNA, the percentage of samples with detectable plasma HIV RNA increased significantly (*p* = 0.0340, [Chi squared test for trend], Fig. 4c), however, the individual pairwise tests were not statisctically significant (*p* > 0.05, [Fisher's exact test], Fig. 4c). We next compared the level of MS RNA in samples where plasma HIV RNA was detected or not detected using all trial samples including baseline and following panobinostat treatment. We found higher levels of MS RNA in samples which had detectable plasma HIV RNA (*p* = 0.0012, [Mann–Whitney test], Fig. 4d). These data suggest both a qualitative and quantitative relationship between MS RNA and detection of plasma HIV RNA *in vivo*, similar to what we previously described with US RNA and plasma HIV RNA [36].

## 4 Discussion

**Fig. 5:**
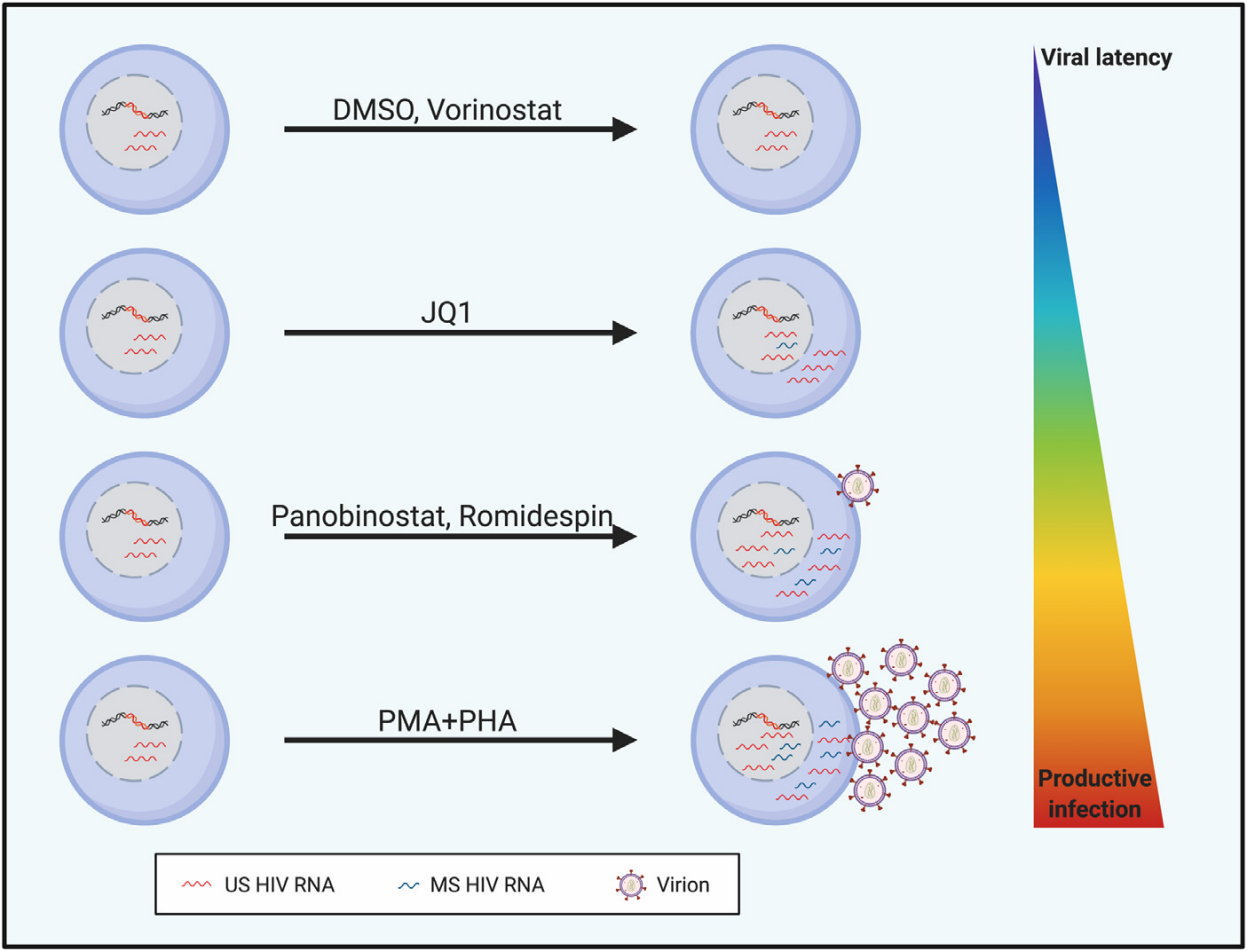
**Proposed model of the impact of LRAs with varying potency on virus transcripts.** Latency reversal can be quantified *ex vivo* and *in vivo* by measuring initiation of transcription (unspliced, US RNA), spliced transcripts (multiply spliced, MS) or release of virions (supernatant or plasma HIV RNA). We show that some LRAs are only able to induce initiation of transcription, while others can induce MS RNA and virion production. *Ex vivo* using total or resting CD4+ T cells from people living with HIV on suppressive antiretroviral therapy, the fold increase in MS RNA and SN RNA was highly correlated. PMA+PHA induced the largest fold increase in all forms of HIV RNA likely because the stimulus potently induces all stages of viral production as well as cellular proliferation. This figure was created using BioRender.com.

In this study, we showed that there was a high level of constitutive expression of US RNA in both total and resting CD4+ T cells in PLWH on long-term ART. The level of baseline MS RNA was low to undetectable in both total and resting CD4+ T cells. The primers used to detect MS RNA bind to regions of the virus which are commonly defective and therefore detection of MS RNA is not only a reflection of viral transcription but possibly also the frequency of intact virus. Following stimulation of CD4+ T cells collected from PLWH on ART, we found that all LRAs led to an increase in US RNA except for vorinostat, but only PMA+PHA and to a lesser extent romidepsin and panobinostat led to increases in MS RNA (Fig. 5). The change in US RNA following LRA treatment was strongly associated with basal or constitutive expression of US RNA. Finally, we identified a strong correlation in the fold increase in MS RNA (but not fold increase in US RNA) with the fold increase in SN RNA. Our findings suggest that initiation of transcription may occur relatively easily in most cells and is related to constitutive levels of US RNA but production of MS RNA and subsequent SN RNA is a rarer event, requiring both intact viral sequences and efficient activation of both transcription and translation.

Latently infected cells have classically been described as containing integrated HIV DNA while being transcriptionally silent and therefore remaining invisible to the host's immune system [49]. However, many studies have clearly shown that US RNA is consistently detected *in vivo* in PLWH on ART prior to any clinical intervention. It has recently been shown that despite consistent detection of US RNA *in vivo* or *ex vivo* on a population level in the absence of any intervention, >90% of CD4+ T cells are transcriptionally silent on the single cell level [50,51], consistent with only a subset of infected cells being constitutively transcriptionally active. We propose that baseline measurement of US RNA will include the detection of incomplete transcripts, consistent with transcriptional initiation but downstream blocks to virus production, as recently described by others [18]. In addition, these transcripts could also arise from both intact and defective proviruses where there are downstream deletions or stop codons [41,43,44,46,50].

Based on our primer sequence analysis, we found that the primers used to detect US RNA would detect close to 40% of defective proviruses, while primers to detect MS RNA would only detect 8% of defective proviruses. Given that the nFLS database is derived from DNA sequencing and not RNA transcript sequencing and that we could not assess the binding of the first forward primer for US RNA that binds to the 5’LTR, this is likely an overestimation given that the generation of MS RNA requires *cis*-acting sequences within the HIV genome that must be intact [52]. In summary, we estimate that a very small subset of infected cells from PLWH on ART are able to produce MS RNA, either constitutively or following treatment with an LRA.

We found a positive relationship between constitutive expression of US RNA and the increase in US RNA following stimulation. This was consistent with our previous observations in a clinical trial of vorinostat in PLWH on ART. We previously described that following vorinostat treatment, the fold change in US RNA was correlated with baseline US RNA [17]. We also found in a clinical trial of disulfiram treatment in PLWH, individuals with high baseline US RNA had statistically significant increases in US RNA post-disulfiram treatment [27]. In other words, when measuring US RNA, an increase in US RNA following an LRA was dependent on both the basal transcriptional state of the virus in a cell and the potency of the intervention. Here we showed that an increase in US RNA was commonly induced by nearly all LRAs *ex vivo*, but production of MS RNA and SN RNA, which is more relevant for ultimate clearance of infected cells, was only observed following stimulation with PMA+PHA and some HDACi. Therefore, measurement of MS RNA and either SN RNA (*in vitro*) or plasma HIV RNA (*in vivo*) are more relevant markers of latency reversal.

We found less efficient generation and accumulation of MS RNA and virion production with all LRAs compared to PMA+PHA. In contrast to other reports [18], we detected MS RNA production with the HDACi romidepsin and panobinostat, but to a lesser extent compared to stimulation with PMA+PHA. One potential explanation for these findings could be the effects of each of these stimuli on cellular proliferation and survival. PMA+PHA will significantly drive cellular proliferation [53] and therefore allow for survival of cells that may be producing virions. In contrast, activation with an HDACi, particularly romidepsin and panobinostat, is associated with enhanced cell death and lack of cellular proliferation (see figure 5 for proposed model).

Our findings are consistent with a previous report which investigated the effects of stimulation of total CD4+ T cells with full T cell activation using plate-coated anti-CD3/CD28 antibodies [54]. The authors found that cell-associated MS RNA correlated with cell-free HIV RNA, whereas cell-associated US RNA did not [54]. Furthermore, they showed a positive correlation between cell-free HIV RNA and viral infectivity as measured by a modified viral outgrowth assay consistent with MS HIV RNA more likely reflecting changes in replication-competent virus compared to US RNA. A key difference from our work is that we evaluated the relative production of US, MS and supernatant RNA following stimulation with LRAs, not full T cell activation, which will also drive cellular proliferation and many other changes. In addition, we examined both total and resting CD4+ T cells and we confirmed our findings *in vivo* using results from a clinical trial of the LRA panobinostat. We also showed that the likelihood of a defective virus producing US RNA compared to MS RNA is far greater, based on a published large sequence database of defective viruses. It is important to note that *in vitro* defective proviruses can still produce spliced RNA transcripts and proteins [55,56] which can be recognized by cytotoxic T lymphocytes [55]. Given that the overall goal of latency reversal is to eliminate intact replication-competent virus and given the complexity of viral outgrowth assays and full-length virus sequencing, we conclude that quantification of MS RNA is a far more practical assay to use in clinical trials and *ex vivo* screening of LRAs.

We did not assess the contribution of read-through transcripts to the pool of US HIV RNA. As HIV tends to integrate into actively transcribing genes, HIV sequences can be generated by transcription from a host promoter and contain LTR-gag sequences that would appear to be LTR-driven US HIV RNA by qPCR. It has previously been shown that readthrough transcripts are detected in PLWH on ART and can be increased following *ex vivo* stimulation with LRAs [20,57,58]. Therefore, quantifying US HIV RNA alone may also overestimate the true level of HIV-specific latency reversal as it can include non-specific host-gene transcription.

Throughout this study, we performed parallel analyses of total and resting CD4+ T cells to evaluate the response to LRAs. While HIV latency was first described in resting CD4+ T cells, it has since been well described that HIV can persist on ART in many T cell subsets, including activated and proliferating CD4+ T cells [2,59], which are removed when resting CD4+ T cells are purified. Here, we found that the response to LRAs was similar between total and resting CD4+ T cells. Interestingly, we found that total compared to resting CD4+ T cells had higher levels of HIV DNA and US HIV RNA, consistent with persistence of HIV infection in cells other than resting CD4+ T cells in PLWH on ART.

In conclusion, there is a clinical need for high throughput assays to determine whether an LRA can effectively reverse HIV latency by activating intact replication-competent virus. To date, most clinical trials of latency reversing agents have relied on demonstrating an increase in US RNA [17,[21], [22], [23],^27^,^60^,^61^]. Here, we show that production of virions *ex vivo* and *in vivo* (measured as an increase in supernatant or plasma HIV RNA, respectively) best correlates with changes in MS RNA. Even though defective virus can indeed produce viral proteins [55], given the main purpose of latency reversal is to eliminate cells infected with intact replication-competent virus and given the high frequency of defective virus in PLWH on ART, our findings support the use of MS RNA rather than US RNA as a better indicator of meaningful latency reversal. Assessing changes in MS RNA should be incorporated in the pre-clinical and clinical evaluation of new LRAs.

## Contributors

Conceptualization, JMZ, GK, DFJP, and SRL. Methodology, JMZ, GK, WZ and JLA. Investigation, JMZ, GK, WZ, AR, MJG, RDP, MR, AD, MG, JLA. Resources, SGD, JM, TAR. Laboratory experimental analyses, JMZ, MJG, MR, SRL; Statistical analyses JMZ, PB and SRL. Writing - original draft, JMZ, GK and SRL. Writing – Review & Editing, All authors read and approved the final draft and have had access to the raw data. JMZ and SRL can verify the accuracy of all raw data in this study. Funding acquisition, SRL, DFJP.

## Declaration of Interests

SRL's institution has received funding from the National Health and Medical Research Council (NHMRC) of Australia, National Institutes for Health, American Foundation for AIDS Research; Merck, ViiV and Gilead for investigator-initiated research; Merck, ViiV and Gilead for educational activities. She is on the advisory board of Abivax and Innivirax.

## Appendix Supplementary materials

Image, application 1
