# Supplementary material for: Multiply spliced HIV RNA is a predictive measure of virus production ex vivo and in vivo following reversal of HIV latency
Source: eBioMedicine. 2021 Feb 26;65:103241. doi: 10.1016/j.ebiom.2021.103241 (PMC7920823; doi:10.1016/j.ebiom.2021.103241)
Supplement: Supplementary file 1 [file mmc1.docx]

**Supplementary Figure 1:** Toxicity and proliferation following treatment with LRAs in total and resting CD4+ T cells. (**a**) Representative flow gating strategy of total CD4+ T cells from one donor at day 3 post-treatment is shown. Cell viability following treatment with each LRA was quantified by flow cytometry using a live/dead stain following treatment with each LRA in total (**b**) and resting (**c**) CD4+ T cells at days 1, 3, and 6. Proliferation was measured by expression of Ki-67 following treatment with each LRA in total (**d**) and resting (**e**) CD4+ T cells at days 1, 3, and 6. Data are presented as the mean +/- SEM of three donors performed in duplicate.

**Supplementary Figure 2: US, MS and SN HIV RNA in total and resting CD4+ T cells from PLWH on ART 3 days following stimulation with LRAs *ex vivo*. Related to Figure 2.** (**a**) US and (**b**) MS RNA were quantified in total and resting CD4+ T cells and (**c**) SN RNA was quantified in culture supernatant following stimulation with different LRAs for 3 days. Absolute values are shown as the mean +/- SEM. Each dot represents a single donor. p values were calculated using a paired t test on logarithmically transformed data comparing all LRA values to the DMSO control. *p < 0.05; **p < 0.01; ***p < 0.001; ****p < 0.0001.

**Supplemental Figure 3:** **Correlation between US or MS RNA at baseline and 3 days post-stimulation.** Pearson correlation efficient and 95% confidence intervals are shown for comparisons between (**a**) US RNA and (**b**) MS RNA at baseline and at 3 days post-stimulation with each LRA. (**c**) Unadjusted Pearson correlation plots are shown for either US RNA or MS RNA at baseline and at day 3 following stimulation in total (closed) and resting (open) CD4+ T cells. The Pearson correlation coefficients (r) and p values are shown. Each donor is shown as a different symbol and each LRA a different color. *p < 0.05; **p < 0.01; ***p < 0.001, ****p < 0.0001.

**Tables**

**Supplementary Table 1:** Participant demographics

| **Participant ID** | **Age (years)** | **Sex (M or F)** | **Duration on ART (years)** | **CD4 T cell count (cells/µL)** | **Viral load (copies/mL)** |
| --- | --- | --- | --- | --- | --- |
| 1 | 45 | M | 5.9 | 722 | < 20 |
| 2 | 65 | M | 18.8 | 767 | < 20 |
| 3 | 59 | M | 20.5 | 280 | < 20 |
| 4 | 51 | M | 18.9 | 372 | < 20 |
| 5 | 63 | M | 13.2 | 463 | < 20 |
| 6 | 60 | M | 13.3 | 577 | < 20 |
| 7 | 57 | M | 14.7 | 744 | < 20 |
| **Median** | 59 |  | 14.7 | 577 | < 20 |

**Supplementary Table 2:** Primer sequences for US and MS HIV RNA qRT-PCR.

| **Primer** | **Sequence** | **Reference** |
| --- | --- | --- |
| US RNA forward 1^st^ round (MH535) | 5’ - AACTAGGGAACCCACTGCTTAAG-3’ | (61) |
| US RNA reverse 1^st^ round (SL20) | 5' - TCTCCTTCTAGCCTCCGCTAGTC-3' | (62) |
| US RNA forward 2^nd^ round (SL19) | 5’ - TCTCTAGCAGTGGCGCCCGAACA-3’ | (62) |
| US RNA reverse 2^nd^ round (SL20) | 5’ - TCTCCTTCTAGCCTCCGCTAGTC-3’ | (62) |
| MS RNA forward 1^st^ round (SL28) | 5' - CTTAGGCATCTCCTATGGCAGGAA-3' | (62) |
| MS RNA reverse 1^st^ round (TM1) | 5’ - TCAAGCGGTGGTAGCTGAAGAGG-3’ | (36) |
| MS RNA forward 2^nd^ round (SL28) | 5’ - CTTAGGCATCTCCTATGGCAGGAA-3’ | (62) |
| MS RNA reverse 2^nd^ round (SL29) | 5’ - TTCCTTCGGGCCTGTCGGGTCCC-3’ | (62) |

**Supplementary Table 3:** Primer sequences for total HIV DNA and CCR5 qPCR.

| **Primer** | **Sequence** | **Reference** |
| --- | --- | --- |
| Total HIV DNA forward (SL19) | 5’ – TCTCTAGCAGTGGCGCCCGAACA – 3’ | (63) |
| Total HIV DNA reverse (SL20) | 5’ – TCTCCTTCTAGCCTCCGCTAGTC – 3’ | (63) |
| Total HIV DNA probe (SL30) | 5' –CGGGAGTACTCACCAGTCGCCGCCCCTCGCCCTCCCG – 3’ | (16) |
| CCR5 forward | 5’ – GCTGTGTTTGCGTCTCTCCCAGGA – 3’ | (39) |
| CCR5 reverse | 5’ – CTCACAGCCCTGTGCCTCTTCTTC – 3’ | (39) |

**Supplemental Figure 1**

**Supplemental Figure 2**

**
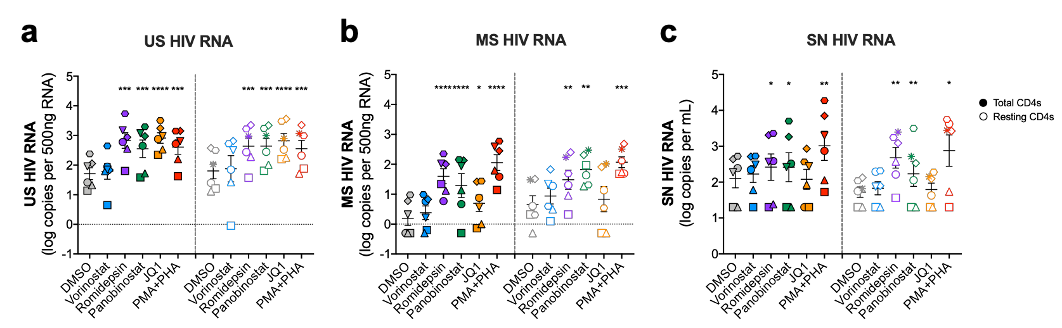
**

**Supplemental Figure 3**

**
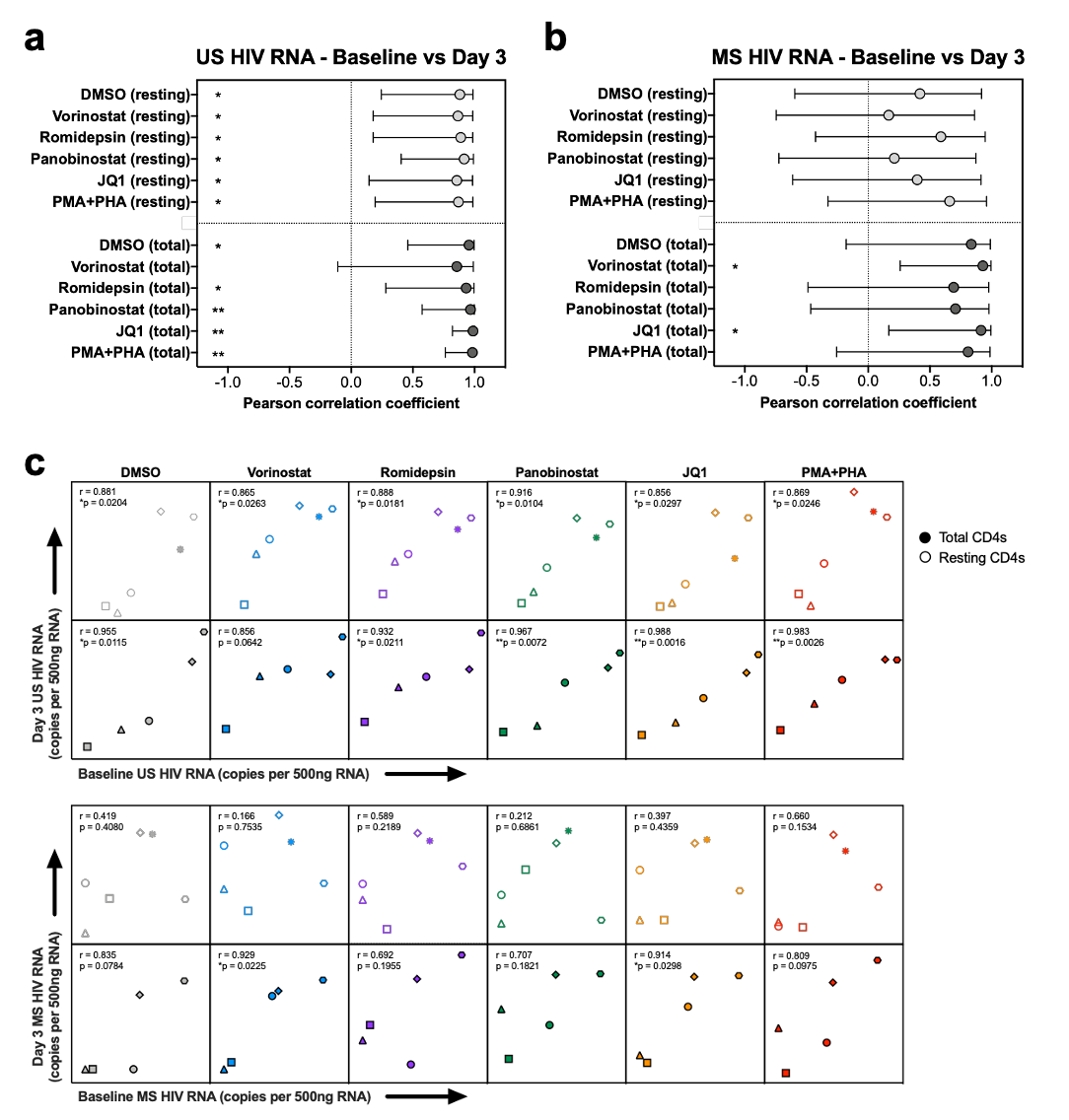
**

**References**

61. Butler SL, Hansen MS, Bushman FD. A quantitative assay for HIV DNA integration in vivo. Nat Med. 2001;7(5):631-4.

62. Saksela K, Muchmore E, Girard M, Fultz P, Baltimore D. High viral load in lymph nodes and latent human immunodeficiency virus (HIV) in peripheral blood cells of HIV-1-infected chimpanzees. J Virol. 1993;67(12):7423-7.

63. Vesanen M, Markowitz M, Cao Y, Ho DD, Saksela K. Human immunodeficiency virus type-1 mRNA splicing pattern in infected persons is determined by the proportion of newly infected cells. Virology. 1997;236(1):104-9.
